# Designing a clustering algorithm for optimizing health station locations

**DOI:** 10.1186/s12942-025-00390-1

**Published:** 2025-03-22

**Authors:** Pasi Fränti, Sami Sieranoja, Tiina Laatikainen

**Affiliations:** 1 Machine Learning Group, School of Computing, University of Eastern Finland, P.O. Box 111, 80101 Joensuu, Finland; 2 Institute of Public Health and Clinical Nutrition, University of Eastern Finland, P.O. Box 1627, 70211 Kuopio, Finland; 3 Joint Municipal Authority for North Karelia Social and Health Services (Siun sote), Tikkamäentie 16, 80210 Joensuu, Finland

**Keywords:** Facility location, Health care optimization, Clustering, Maximum coverage, Random swap

## Abstract

In this paper, we define the optimization of health station locations as a clustering problem. We design a robust algorithm for the problem using a pre-calculated overhead graph for fast distance calculations and apply a robust clustering algorithm called random swap to provide accurate optimization results. We study the effect of three cost functions (Euclidean distance, squared Euclidean distance, travel cost) using real patient locations in North Karelia, Finland. We compare the optimization results with the existing health station locations. We found that the algorithm optimized the locations beyond administrative borders and strongly utilized the transport network. The results can provide additional insight for the decision-makers.

## Introduction

At present, there is substantial pressure to reduce healthcare service costs by enhancing the optimization of healthcare services, potentially resulting in a reduction of the existing healthcare station network and the services they offer. Yet, a high quality of health care services would lead to healthier citizens and, in this way, reduce the overall demand for these services.

Accessibility of the service is one factor in this optimization. First, it can improve early diagnoses and treatments and support the better provision of preventive health care [1]. Accessibility is a major determinant of participation in the service, according to Gu et al. [2]. Geographic distance to services has been identified as a significant barrier to regular checkup visits and chronic care visits, especially in rural areas, whereas acute care visits seem to be less sensitive to distance [3]. In Finland, among patients with mental health problems, distance was negatively associated both with in-person visits to health stations as well as in-home visits [4].

Second, good accessibility can save lives in case of emergency situations and prevent long-term consequences caused by delayed treatment. Patients living closer to a percutaneous coronary intervention (PCI) capable cardiac unit have a higher chance of survival than those who live far away, according to Di Domenicantonio et al. [5].

Third, improving accessibility can reduce the travel costs of the patient, both direct costs and time loss associated with indirect costs, relieving the financial burden for the patients and lowering the threshold to seek preventive care. In addition to costs for patients, long distances create societal costs, for example, in different forms of reimbursements and transport costs [6]. Accessibility is also a question of equity and fairness of service provision that should be considered [7].

Healthcare accessibility has been modeled as *a maximum coverage location* problem by maximizing the number of patients reached given some distance threshold [8]. The results in [9], however, suggested that maximizing coverage (minimizing the patients at risk) leads to a significant increase in the average travel time of the patients. Burkey et al. [10] reported similar results, optimization for the coverage reduced the patients at risk from 6.9 to 2.7%, on average, in the case of health care services in three US states (North Carolina, South Carolina, and Virginia), but it increased the average travel time by 13%.

Wang & Tang [11] proposed to minimize the variance of the distance or travel time for equal accessibility. Accessibility to tertiary and secondary facilities was studied in [12] using data from Shenzhen, China. The travel time was estimated by the Baidu Map application programming interface (API), and optimization was performed using the particle swarm optimization (PSO) algorithm [13].

Burkey et al. [10] reported that the existing locations already provide near-optimal geographic access to healthcare services in three US states (North Carolina, South Carolina, and Virginia). The average travel time of the existing facilities could be further reduced by only about 5% by the -p-median clustering algorithm. The only exception was Tennessee, where the reduction was 15%. In the case of myocardial infarction patients in Finland, better optimization could decrease the average travel time to the hospital by only 3.7% [9]. A more remarkable saving was reported by Gu et al. [2], who managed to increase the accessibility of breast cancer screening services by 14% (from 0.35 to 0.40). They used Google Maps API to estimate the travel distance and time. An even more significant reduction in distance (33%) was reported by Fo & Silva Mota [14] with data from the Sao Paulo metropolitan area.

A completely different approach to measuring accessibility is the *two-step floating catchment area* (2SFCA) method by Shen et al. [15], and its hierarchical variants [16, 17]. They measure the ratio of primary-care physicians to population. It first counts the ratio of physicians to their surrounding population (within a given travel time) and then sums up the ratios around the demand locations. This leads to a different optimization problem, which would close to resemble maximum coverage but favor bigger units. Accordingly, Tao et al. [17] observed that the healthcare facilities in Shenzhen are unevenly distributed due to the concentrated distribution of tertiary hospitals.

The use of a clustering algorithm can find theoretically better facility locations. However, existing research suggests that optimizing for one criterion, like coverage, can lead to inferior optimization for another criterion, like average travel time. The clustering process itself also includes several design parameters like the choice of the algorithm and the estimation of the travel time, which both may have a significant effect on the optimization result. It is an open question how these design choices affect the clustering results.

To address these questions, we perform an experimental study with a sample of diabetes-related healthcare visits from SiunSote in North Karelia, Finland, between 2011 and 2014. Instead of classical k-means or its variant p-median, we use a more robust clustering algorithm to reduce the effect of algorithm artifacts. We then show the optimization results with different criteria, including Euclidean distance, squared Euclidean, travel time, and travel cost. We also consider the efficiency of the optimization process.

We also analyze the optimization results. While the results are based on a selected sub-sample of patient data and cannot be used to guide the healthcare organization, they reveal several interesting facts and trends in the area. For example, the location of existing healthcare stations and allocation of patients to these follow municipal borders, whereas the algorithm does not have such a border as it has no restrictions to allocate patients to nearby health stations in the neighboring municipality.

The results also have relevance to other healthcare services. For example, the nurse districting problem in [18] clusters the patients by simple k-means and tabu search based on their locations. The home care scheduling problem has been considered a two-objective optimization problem, which aims at minimizing operating costs while maximizing the quality of service at the same time [19]. Other closely related problems include ambulance location and relocation problems.

## Clustering

Next, we describe the clustering component, what components it includes, and explain the choices behind each of them. The clustering is integrated in a Web-tool described in [20].

### Distance calculation

K-means is the most common clustering algorithm. It minimizes squared Euclidean distances between the data (patient locations) and their nearest centroid. Squared distance is widely used even if it does not inherently correspond to real-world geographic phenomena. Some attempts have been made to make the connection, though. For example, Zhou and Li [21] modeled the cost of disaster losses as a quadratic function of the distance from the emergency facility to the disaster location.

Another common approach is to maximize the number of locations that are within a given distance from its nearest facility. Church [22] defined this as *a maximal covering location problem*. It is also referred to as *threshold distance* [23], where the distance cost is 0 if the distance to the centroid is less than a predefined threshold value, otherwise, it is 1. Fränti et al. [24] minimized the number of myocardial infarction patients at risk by defining at-risk individuals based on their proximity to the nearest percutaneous coronary intervention (PCI)-capable hospital, specifically considering whether a patient resides beyond a predetermined time limit.

Absolute (non-squared) distance is the most used and natural choice in the case of location-based applications. It has a slight difference from its squared variant in the Euclidean distance case. The optimal centroid location of a cluster is its geometric center in the case of squared distance but its median in the case of absolute distance. The average (or median) can be calculated for each coordinate independently. The median is also known as the spatial median [25], and the corresponding k-means variant is known as the *p-median* [26, 27].

Simple Euclidean distance, however, was shown to cause bias in the facility optimization [24]. For this reason, travel distance (or time) is recommended. Calculating the shortest distance via road network is straightforward but requires lots of computation. A single shortest path calculation is fast, but facility optimization requires millions of such calculations. The locations of the stations are also dynamically changing during the optimization process, which prevents the use of a pre-calculated distance matrix. The consequence is that the optimization processing may take even days.

**Fig. 1:**
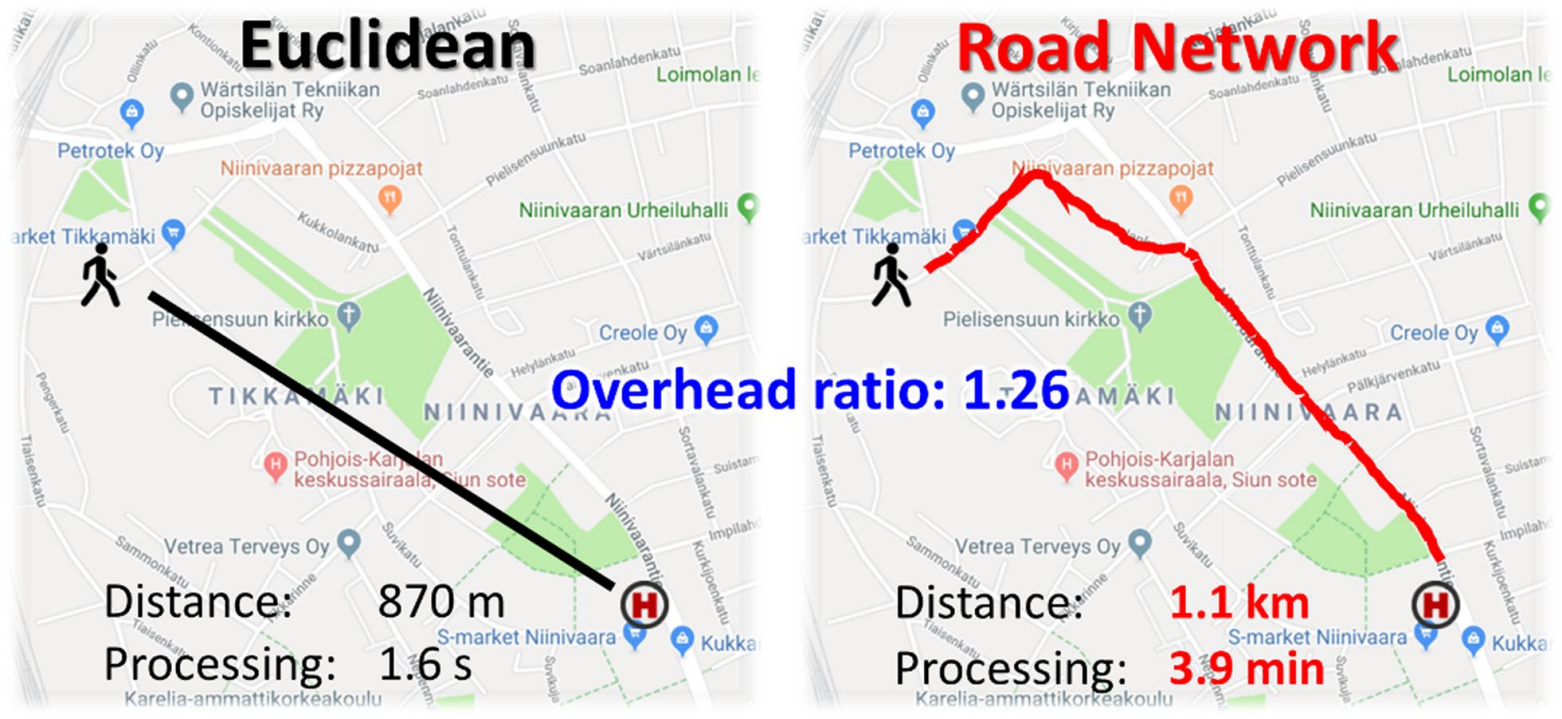
Example of the detour index between Euclidean and road network distances from the patient location (Huvilakatu) to the nearest health station (Suvikatu station)

Boscoe [28] showed that Euclidean distance is an adequate approximation for travel distance in the United States when multiplied by a constant factor of 1.4. They call this factor *the detour index*. It virtually equals the diagonal of a unit square corresponding to the Manhattan distance. However, our data is mostly in areas where lakes and rivers make the road network more complex, and a simple Manhattan distance would not be accurate. In city areas, the factor can be smaller than 1.4 (see Fig. 1), but in areas containing rivers and lakes, the factor can be much bigger. In general, there are large differences.

Mariescu-Istodor and Fränti [29] adapted this detour index locally by constructing a so-called *overhead graph*. The graph was built as pre-processing using patient locations in the data and the road network to identify traffic points. These points constitute the nodes of the graph. *The overhead ratio* was then calculated for all pairs of nodes in the graph as the ratio of their true travel distance and the corresponding Euclidean distance. The larger the overhead value, the longer the detour. The values are stored in the graph (represented by a matrix) and used in the optimization process.

During the optimization, when we need to estimate the travel distance between a patient’s location and a given health station, we first calculate their Euclidean distance. We then find the nearest graph nodes of these two locations and obtain the stored overhead ratio between these two locations. The Euclidean distance is then multiplied by this constant to obtain an estimation of the travel distance. Travel times are derived directly from the travel distances using the average speed of each road segment. Dynamics like rush hours are not considered, and optimization is made merely to minimize the average.

**Fig. 2:**
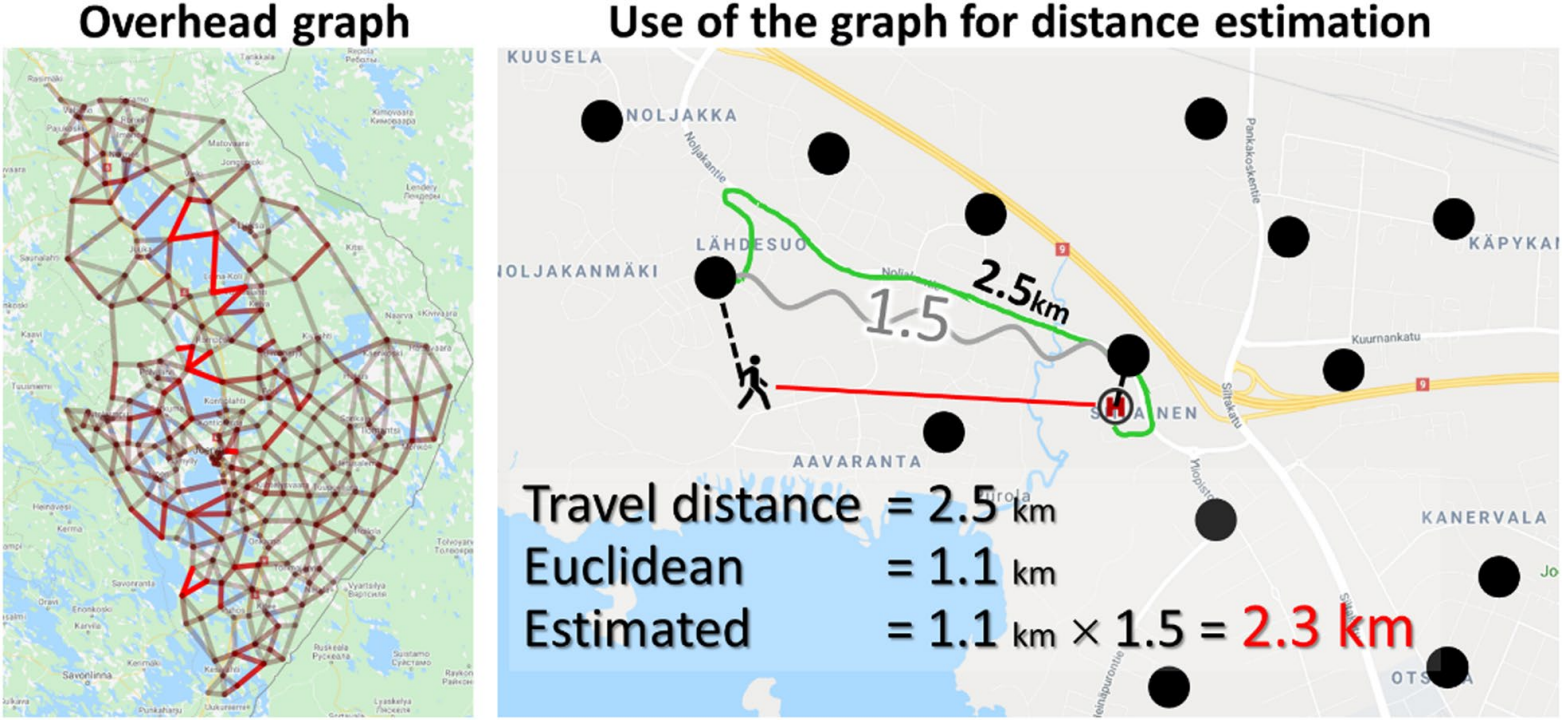
Overhead graph with 256 nodes constructed for the North Karelia region and an example of its use

This process is extremely efficient, requiring only a single lookup table and one multiplication operation (overhead ratio × Euclidean distance). With our data, this reduces the processing time from 2 weeks to about 15 min when using 10,000 iterations of the clustering algorithm. The huge speed-up is achieved at the cost of minor inaccuracy in the distance estimation, 2%, according to [29], and with additional memory of 512 × 512 = 0.25 MB for storing the lookup table. The process with two sample graphs is shown in Fig. 2.

### Optimization function

K-means is by far the most common clustering algorithm and would be the most obvious choice for use here as well. However, it minimizes the sum of squared Euclidean distances, and it is unclear whether geographical distances should be squared in this application. A more common choice is to calculate the sum of Euclidean distances as such (without squaring). However, neither of these satisfied our needs as we are also interested in the travel costs of the patients. We, therefore, consider also the sum of the travel costs as the optimization function.

The three optimization functions can be written mathematically as follows:

$$\text{Euclidean}: {\sum }_{p\in {\text{patients}}}{d}_{L2}\left(p,\underset{h\in {\text{HS}}}{\text{argmin }}{d}_{L2}\left(h,p\right)\right)$$

$$\text{Squared Euclidean}: {\sum }_{p\in {\text{patients}}}{d}_{L2}{\left(p,\underset{h\in {\text{HS}}}{\text{argmin }}{d}_{L2}{\left(h,p\right)}^{2}\right)}^{2}$$

$$\text{Travel cost}: {\sum }_{p\in {\text{patients}}}{d}_{travel}\left(p,\underset{h\in {\text{HS}}}{\text{argmin }}{d}_{travel}\left(h,p\right)\right)$$

where *HS* refers to the health stations.

For estimating the travel costs, we adopt the cost model presented by [30] tailored for the local region. The model assumes that patients use the bus when the distance to the nearest bus stop is less than 200 m, otherwise, own car is used. Exceptions are patients living within 1 km from the hospital who are expected to walk to the health station with 0 € cost. People 80 years or older are assumed to use taxis.

The model's key elements are summarized in Table 1. Our model is slightly simplified; instead of considering different zones for bus fares, we apply a flat rate of 5.1€ per bus trip. Leminen et al. model also considers the cost of the patient's time during travel based on the average hourly gross wage in the respective zip code area. We have omitted this component to streamline the optimization process.

**Table 1:**
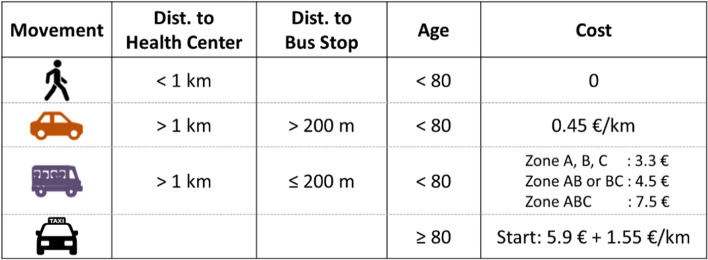
Travel cost model as presented in Leminen et al. [30]

The ideal goal is to maximize accessibility, but it is not clear how to measure it. We use distance, travel time, and travel costs. Travel time is derived directly from travel distance based on the average speeds of the road segments used and is the most obvious measure. However, people also tend to minimize costs in case of non-urgent visits, so the travel cost is also a relevant indicator of accessibility.

### Clustering algorithm

**Fig. 3:**
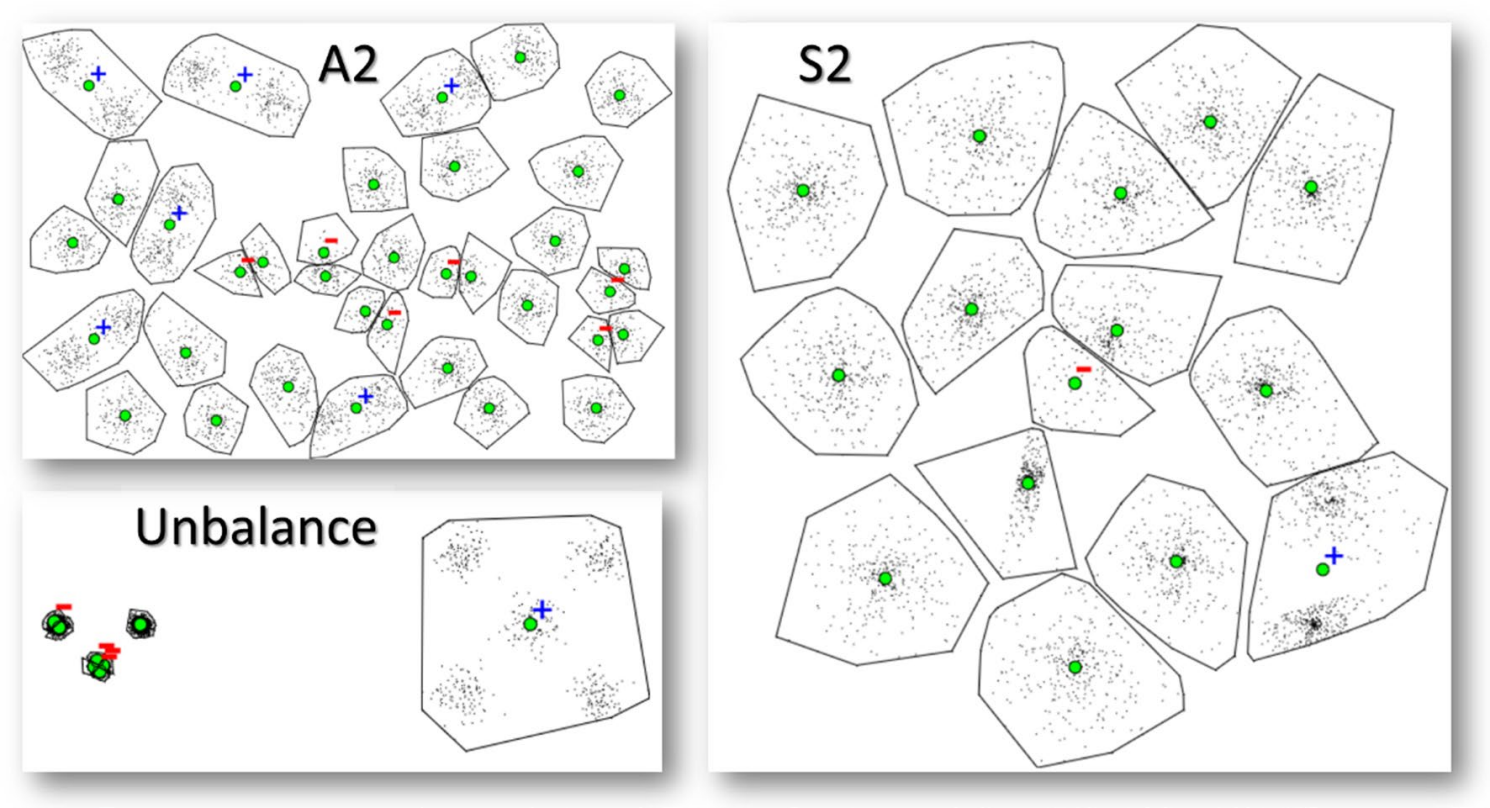
Incorrectly detected clusters (+ sign signifies too many centroids,—sign too few) happen in k-means when there are many clusters and some of them are well separated. K-means may fail even with seemingly easy datasets (named A2, S2, Unbalance) to find all clusters correctly because the algorithm is incapable of moving the centroids across empty areas (deserts). Repeating the algorithm compensates for this only partly but relies too much on luck

K-means and its variants like p-median would be the most obvious choices for the clustering algorithm, but they can be inaccurate, see Fig. 3. It was shown in [31] that k-means works worse when the number of clusters is high, and the clusters are well separated. Part of these problems can be compensated by repeating the algorithm multiple times [32] at the cost of increased processing time or by better initialization using methods like Maxmin [33] or its variant called k-means++ [34]. Despite k-means working well for most data, neither of these alternatives was able to cluster all benchmark datasets correctly [32].

P-median [26, 27] suffers from the same problems as k-means. P-median uses a median for the cluster centroid instead of a mean. It is potentially more robust on noise, but recent results have shown Medoid performing poorly in the context of averaging GPS trajectories [35]. Our data is from patients' real home locations, which is much less noisy than the result of some medical measurement processes.

The accuracy of k-means is usually sufficient when applied as a data processing component within a more complex pattern recognition system. Kinnunen et al. [36] reported that the choice of the algorithm was negligible on the overall speaker recognition performance as long as a reasonably good algorithm was chosen (including repeated k-means). However, clustering is the core component of our analysis, and high accuracy is required to avoid any bias caused by the algorithm. For this reason, we have selected a more robust algorithm.

Many potentially good clustering algorithms exist including Ward's agglomerative clustering method [37], its enhanced variant called iterative shrinking [38], splitting algorithm [39], global k-means [40], and evolutionary algorithms of which the genetic algorithm (GA) [41] and the self-adaptive genetic algorithm (SAGA) [42] have shown to be the most accurate in terms of minimizing the clustering objective function.

**Fig. 4:**
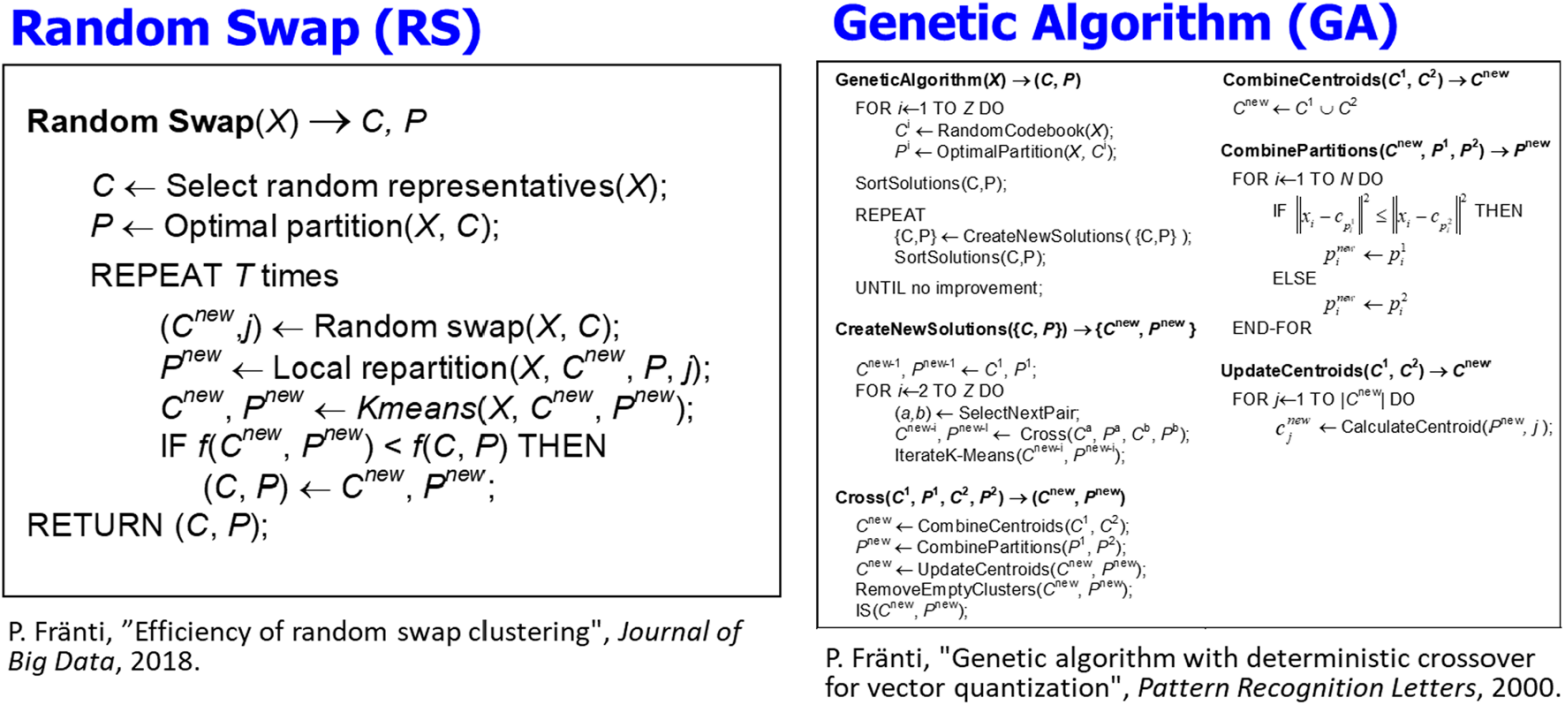
Pseudo codes of two good clustering algorithms. We selected RandoSwap due to its simplicity

Among the many good choices, we select random swap [43]. It performs virtually as well as the more complex genetic algorithms while having the benefit of straightforward implementation, see the pseudo-codes of random swap and the genetic algorithm in Fig. 4. Its simplicity is important because it allows easier adaptation of the algorithm to work with different distance functions such as travel cost. Several implementations with different programming languages^1^ are also publicly available, including a version supporting parallel processing [44].

The random swap algorithm works as follows. It starts with random initial locations for the stations and then uses two k-means iterations to reallocate the patients to their nearest station and then re-optimize the stations' locations. Random swap is a wrapper around the k-means. It selects a random station, relocates it to a new (random) location, and then iterates k-means twice. If the new solution improves the cost function, it is kept; otherwise, the previous solution is restored. While seemingly naïve, this simple trial-and-error approach is effective as the trial swaps can be implemented efficiently.

Here, we use *T* = 5000 trial iterations, which is the original recommendation. The algorithm is not particularly sensitive to this parameter, and the exact value is not even important in offline optimization as we can easily run the algorithm as long as we want, e.g., let the algorithm run 100,000 iterations overnight just to be sure. A theory about how to set this value more accurately can be found in [43]. Visual animation of the algorithm can be found here https://cs.uef.fi/ml/software/ and tutorial here https://www.youtube.com/@pasifranti541.

**Fig. 5:**
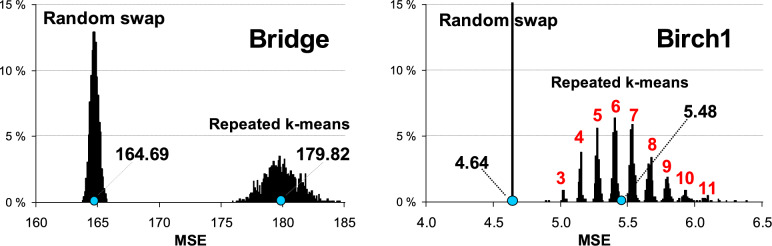
Effect of the optimization by random swap algorithm versus k-means

Figure 5 shows the benefit of random swap over k-means. The average squared distance (MSE) is 10% smaller for data with lots of clusters (Bridge). Repeating k-means 100 times can improve accuracy, but it cannot reach the same accuracy level. For data with 100 separate clusters (Birch1), the difference is 15%. K-means locates 7 clusters wrongly. If repeated 2500 times, the number will be reduced to 3. Even with a small number, it is too much when accuracy is important.

For the centroid, we use arithmetic means of all the patient locations in the cluster. This is not the optimal choice for minimizing the travel cost, but it is the best we can think of, and it can be calculated fast. Finding a better location could theoretically be obtained by a local search around the current location, but re-calculating all distances would be time-consuming. Instead, we consider fine-tuning the centroid location to the nearest existing building. While new buildings can (and probably should) be constructed, this at least restricts the centroids from being located on lakes and inaccessible places without any infrastructure nearby.

## Case study: SiunSote healthcare data

As a case study, we used data from type 2 diabetes patients in North Karelia, Finland. The reason for selecting this cohort is that it includes the exact locations of the patients that we need for optimization. For the full data, we only have the postal code accuracy. It is accurate enough for optimizing the locations of PCI units throughout Finland [24], but it is not necessarily for optimizing the locations of local health stations.

The data is extracted from Siun Sote's electronic patient records, which organizes the regional health services in North Karelia. The data includes all type 2 diabetes patients (n = 9333) diagnosed by the end of 2012 and having visits to primary health care between 2011 and 2014 (175,039 visits in total, averaging 4.7 visits annually per person), see Table 2. Type 2 diabetes patients need frequent follow-up and often have co-morbidities resulting in heavy use of health services. The study restricted the use of primary health care services, and thus, information on specialized care visits was excluded.

**Table 2:** Summary of data

Data	Values
Time range	2011–2014
Cohort	Type 2 Diabetes patients
Number of patients	9333
Male	47% (4387)
Female	53% (4946)
Mean age (in 2011)	67 years
Number of visits	175,039
Geographical location	North-Karelia, Finland

The exact locations were extracted by geocoding the patient home addresses using ArcGIS 10.3 [45]. The information on primary health care visits includes information on the health station where the visit occurred. Their locations were also geocoded based on the address.

The distances to the existing health stations and nearest bus stops were calculated using road network distance information from the OpenStreetMap (OSM) server. Distances to the optimized health station locations were estimated using the overhead graph, as explained in Sect. "Distance calculation". The travel costs were estimated using the model by [30], as discussed in Sect. "Optimization function". It uses factors like patients' age, distance to the health station, and the possibility of using public transport; the most likely travel mode was decided.

## Results

**Fig. 6:**
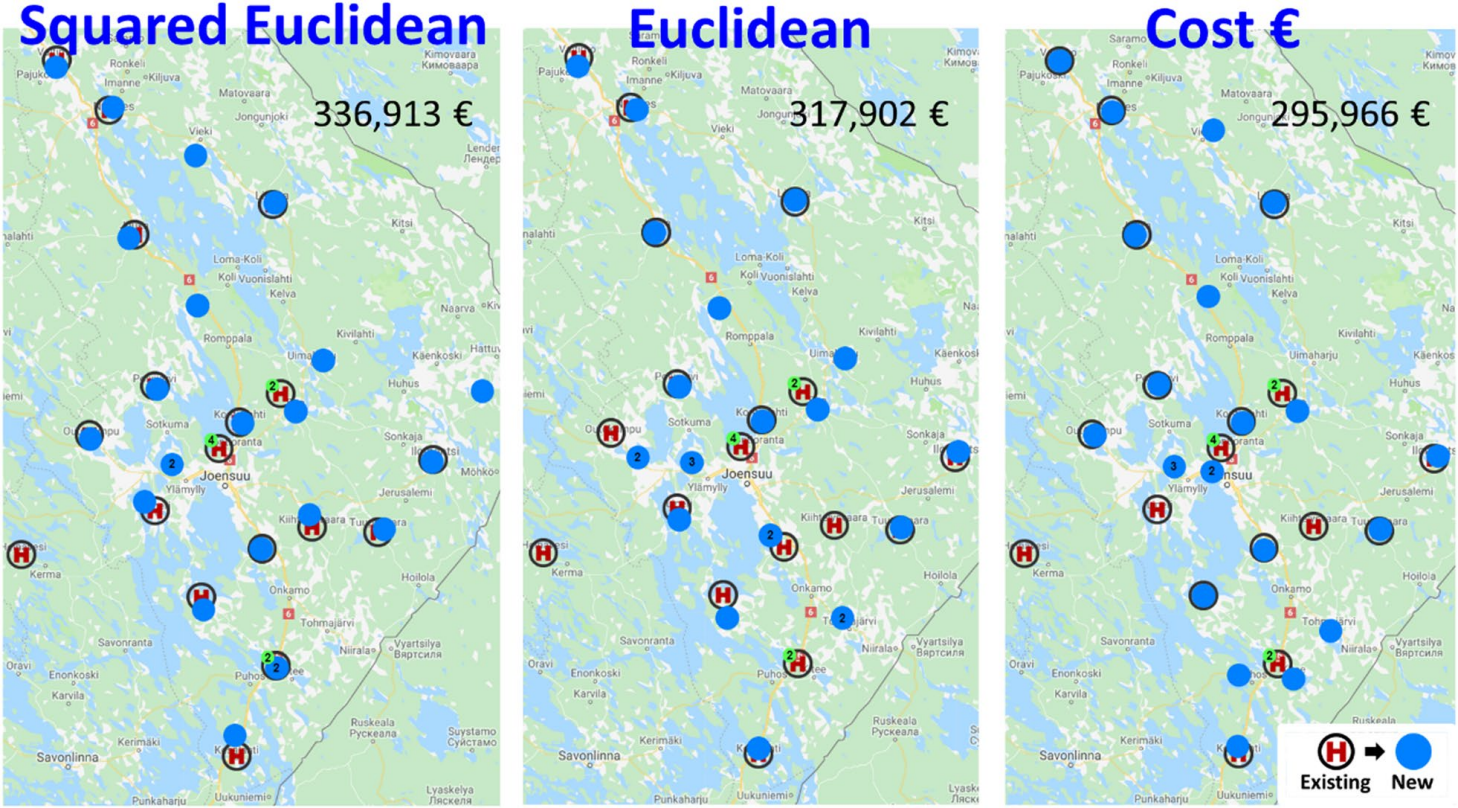
The optimization results are presented for three different distance functions, with the optimized locations indicated by blue points. In some cases, multiple locations are clustered and represented by a single green dot, along with a numerical value denoting the count of stations within the cluster. Additionally, the number above corresponds to the total annual travel cost for all patients, measured in euros

Figure 6 shows the overall optimization results using three alternative distance functions. First, we can see that in all cases, most of the optimized locations are roughly at the same locations as the current health stations, with minor variations. First, squared Euclidean distance is used in standard k-means packages, and it penalizes long distances by using a quadratic function. This creates stations more easily in sparsely inhabited areas. Euclidean distance is more conservative in this regard. The effect of travel cost is more difficult to predict since it utilizes both the road network, the location of bus stops, and the age of the patients (in the case of those older than 80 years).

The total travel cost is the lowest (295 k€/year, 6.76€/visit) when optimized directly for the travel costs. Using Euclidean distance is somewhat worse (317 k€/year, 7.26€/visit) but still slightly better than using squared Euclidean distance (336 k€/year, 7.70€/visit).

Next, we will provide a more detailed discussion of the implications of the optimization results. Out of the 23 stations, 17 have remained near their original locations. These locations benefit from a road network and public transportation system already optimized for their accessibility. However, six health stations were located differently by the algorithm. We will briefly summarize these changes and their underlying reasons as follows:

- Heinävesi removed: lack of patient data.
- Viinijärvi and Ylämylly added: ignoring municipality borders.
- Joensuu Center merged with downtown + Lehmo was removed.
- Uimaharju removed and Kopravaara added: serves more people.
- Mätäsvaara and Multala added: in the middle of nowhere.
- Kiihtelysvaara removed + Rääkkylä relocated logistically.

The first change is merely a data artifact. At the time of data collection, Heinävesi health station belonged to SiunSote (wellbeing services) county but administratively did not belong to the North Karelia province (joined later). As a result, the data had Heinävesi health station but not the patients. For this reason, the algorithm naturally relocated the unused center elsewhere.

**Fig. 7:**
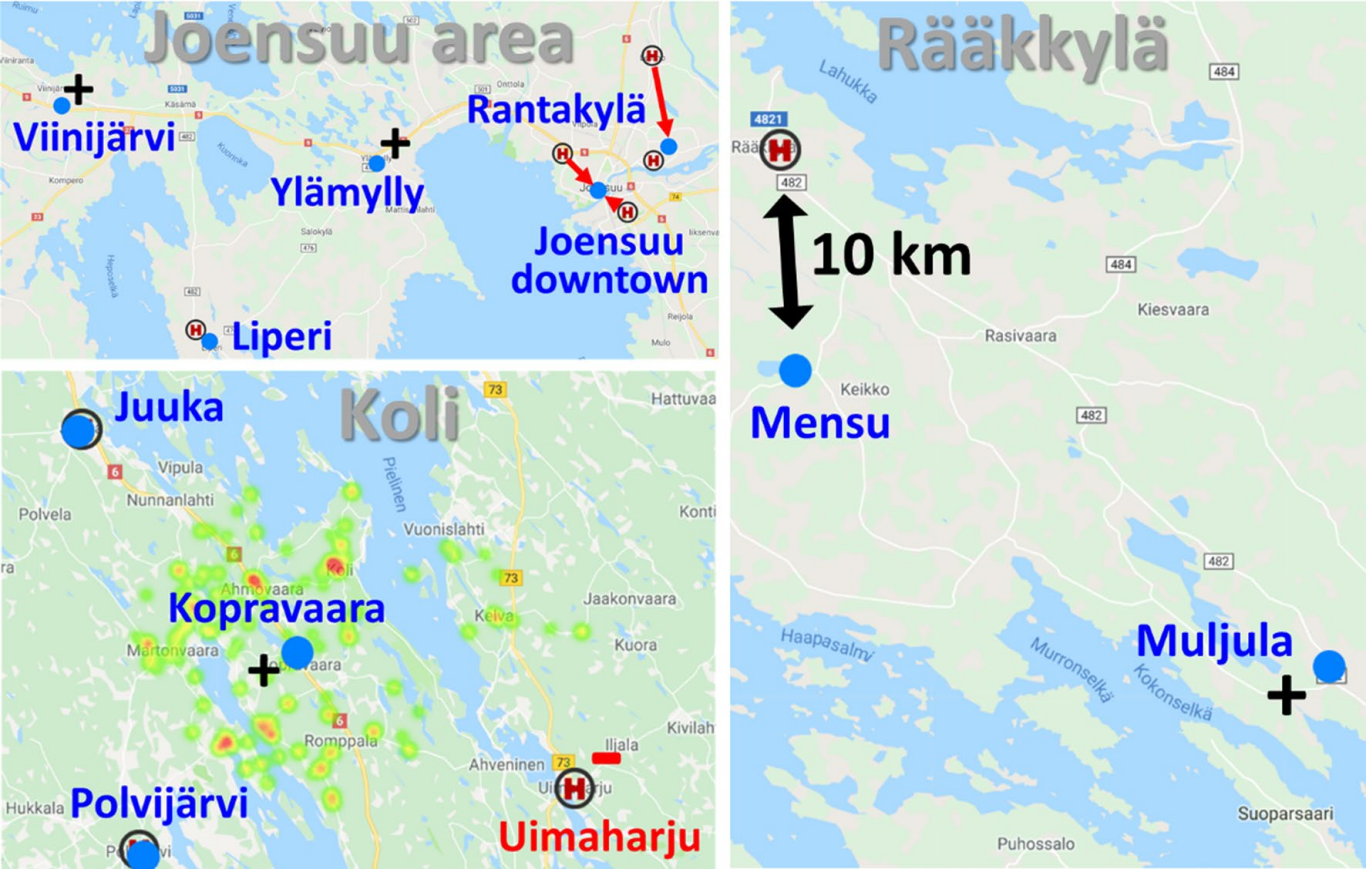
Places with significant changes. The heat map shows the home locations of the patients to whom the new Kopravaara is the nearest health station

The second change is surprising, although logical. The current locations adhere primarily to municipal boundaries. This results in three centers (Siilainen, Niinivaara, Rantakylä) within the Joensuu urban area (~ 59,000 inhabitants), see Fig. 7. In contrast, the neighboring municipality Liperi (12,104 inhabitants) has only one. Liperi itself is an interesting case consisting of three distinct hubs: Liperi center (1,401 inhabitants), Viinijärvi village (746 inhabitants), and Ylämylly suburban center (~ 6,000 inhabitants). Ylämylly is well connected to Joensuu City through a fast motorway and is essentially considered a suburban extension of Joensuu.

The algorithm disregards municipal boundaries and allocates one station to each of the three hubs in Liperi. Placing a station in Ylämylly is logical, as it serves not only Ylämylly but also the adjacent westernmost Joensuu suburban area, Marjala, with a population of 2,328. Relocating one station to Viinijärvi is less obvious but is explained by the significant savings in travel costs. The total cumulative costs of the three new stations are (90 k€) consisting of Liperi (38 k€), Viinijärvi (27 k€), and Ylämylly (25 k€), whereas the cost to the current station in Liperi is 160 k€. This results in cost savings of 70 k€.

The costs of the removed stations in the city of Joensuu are Siilainen (125 k€) and Niinivaara (44 k€), and their replacement at Joensuu downtown is only 139 k€. In other words, the two stations in Joensuu can be simply placed in a better location at the heart of the city downtown, still achieving a 30 k€ reduction in travel costs for the patients. The unused station can then be relocated elsewhere in Ylämylly.

The fourth change is the removal of Uimaharju station and the creation of a new one in Kopravaara, near Koli. Uimaharju is a small town (1,300 inhabitants), best known for its pulp mill and sawmill, but with a steadily decreasing population. Although the removal of the Uimaharju^2^ health station resulted in an increase in travel costs for the Eno health station from 42 to 80 k€, the more strategic placement in the Koli area yielded greater reductions in the overall travel costs for Juuka, Polvijärvi and Kontiolahti. The importance of the Koli area has been increasing due to its famous national park, including the national view of Finland, which has increased both the number of visitors and the population in the area.

**Fig. 8:**
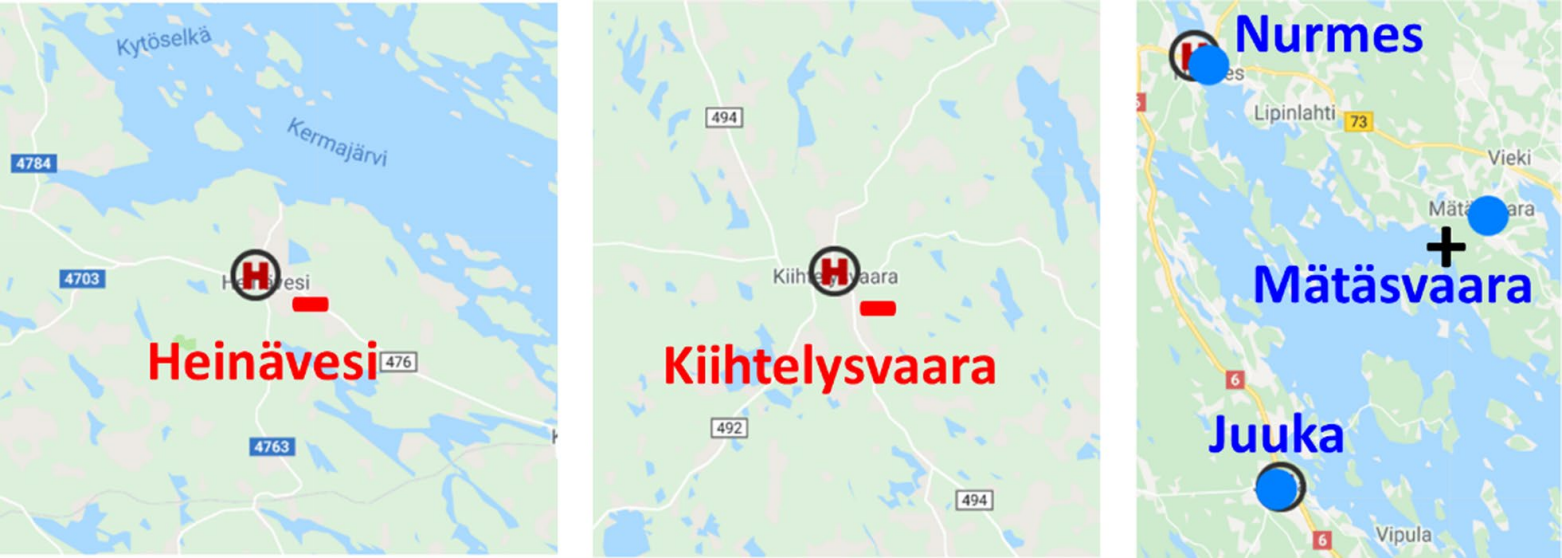
Other places that lost or gained their health station. Heinävesi patient data were missing, so this removal is merely an artifact of the data. Kiihtelysvaara previously operated as a separate municipal station, maintaining its own health station despite serving a relatively small patient population. The algorithm optimized a new station in Mätäsvaara instead

One new addition is Mätäsvaara (917 inhabitants), located roughly halfway between Nurmes and Lieksa, see Fig. 8. Another one is Muljula (382 inhabitants), somewhere between Rääkkylä and Kitee. Neither of them serves a major population, but their effect on travel costs is apparently so high that the model allocated a station there. For example, adding Mätäsvaara reduced the calculative costs of Nurmes from 128 to 88 k€ and Lieksa from 118 to 93 k€.

The removal of Kiihtelysvaara station (3622 inhabitants) did not cause any other changes in the area. The patients are simply treated cost efficiently either in Hammaslahti or Tuupovaara health stations. They all belong to Joensuu municipality.

Rääkkylä is one of the rare tiny municipalities that have kept their independence despite having only about 3,000 inhabitants. It is relatively large (530.85 km^2^) and sparsely populated (4,7 inhabitants per square kilometer), even on a Finland scale. The number of inhabitants per square meter is 9.4 in North Karelia and 17.6 in the entire Finland, on average. Due to the sparsely populated area, the health station is optimized for a logistically better place in Mensu, seemingly in the middle of nowhere, 10 km to the south of the current location in the Rääkkylä town.

To summarize the findings, we can see that the algorithm ignores administrative and historical boundaries and can suggest better locations. Employing three distinct cost functions for optimization, we observed that while squared Euclidean distance (commonly used in standard k-means) enhanced the original locations, the non-squared alternative yielded superior results. The best results came directly from using the travel cost of the patients as an optimization function.

The optimized locations would reduce the total travel costs by 19%, from 367 to 296 k€ (Table 3). This means better accessibility and reducing the average travel time of a single visit from 10.1 to 8.1 min and travel distance from 8.4 to 6.2 km. This would be a remarkable achievement if it could be implemented in real life.

**Table 3:** Effect of the optimization on the average travel distance and travel time

	Optimized for	Travel time	Travel distance	Travel cost
Original	–	10.1 min	8.4 km	367 399 €
Optimized	Squared Euclidean	9.1 min	6.8 km	336 913 €
Optimized	Euclidean	8.4 min	6.2 km	317 902 €
Optimized	Travel cost	8.1 min	6.2 km	295 966 €
Saving		20%	26%	19%

## Conclusions

We have designed a clustering algorithm to optimize the locations of health stations. The advantage of the proposed approach is its simplicity. It should also generalize to entire Finland and other countries if data is available. The travel cost needs to be tuned to the region applied, but the distance and travel time are generic. The clustering approach itself can be tuned relatively easily to be used with other optimization objectives.

The results with SiunSote data in North Karelia, Finland, showed that the algorithm would ignore municipal borders and emphasize the distances and travel costs instead. It allocated a new station in Ylämylly and removed one from Lehmo as there is another station close to Rantakylä, even in a different municipality. The algorithm also removed Uimaharju station, which actually happened in practice after the data was collected. No new station has been founded in the Koli area, though, despite what the algorithm has suggested.

The choice of the cost function has a significant effect on the result. For example, optimizing for squared Euclidean distance (as in standard k-means) would penalize bigger distances more and, therefore, allocate more resources to remote places.

Optimizing travel costs exploits the existing transportation routes. Evidence of this is that the algorithm allocated one station in downtown Joensuu, which is basically next to the start/end points of most bus routes. The travel cost model does not emphasize the distance as much as squared Euclidean, but it also allocates one station halfway between Lieksa and Nurmes, an area having less public transportation and higher travel costs due to the more frequent need for car transportation.

The algorithm can provide better optimization of the resources and would be applicable to data from other areas and different patient cohorts. However, the results cannot be easily applied to real life. Optimizing the overall healthcare service is more complex, and we should also consider factors such as volume, specializations, quality of care, and effectiveness.

The optimization exercise can still provide added value, especially when there is a need to cut down or increase the number of health stations or hospitals that provide different services. Such situations easily occur when health services are reorganized, as happened in Finland at the beginning of 2023 when a large national social and health care reform occurred. The algorithm could also be used for forecasted data of forecasted future population, which would better consider aging and internal migration.

## Limitations

We have done our best to make sure the optimization is well done, and the possibility of results caused by algorithm artifacts remains quite low, contrary to the known limitations of algorithms like k-means and also p-median. However, when dealing with real data, there are always issues that affect the results.

One known limitation in the optimization process is the use of a simple geometric center for the health station location. This may not be the optimal location when minimizing the travel cost. Theoretically, better locations could be found by tuning the locations using an iterative local search algorithm. Starting from the geometric center, the algorithm could consider its neighboring locations in a trial-and-error manner utilizing the overhead graph [29]. However, this would increase the processing time considerably, and it is uncertain if the potentially more accurate optimization would be worth the additional computing.

Another limitation was the lack of Heinävesi population data. The consequence of this is that this particular health station was removed without any cost, and additional resources were placed elsewhere. Another issue is that the volume and specialization of the stations were not considered in the optimization. All health stations were assumed to be of equal importance, having full service for all patients, which is not always the case. Many smaller stations only provide basic primary care services, whereas larger stations can also provide specialized services.

A third limitation of this case study is that only one patient cohort was used: type 2 diabetes patients. This does not, of course, represent the service use of the whole population and is biased toward the elderly population. However, patients with type 2 diabetes are high-service users using primary care services and can be regarded as a typical patient group in primary health care.

Finally, we only considered the geographic distance to measure accessibility. It would be interesting to repeat the optimization process with other objectives and more parameters. For example, the ratio of physicians to patients is an interesting measure, but the simplistic thresholding in the method should be removed; otherwise, it would suffer the same problem as all other methods optimizing maximum coverage.

## Data Availability

No datasets were generated or analysed during the current study.

## Footnotes

1 https://cs.uef.fi/ml/software/ and https://github.com/uef-machine-learning

2 Uimaharju health station was stopped in 2019. No new station was founded in Koli, though.
